# The application of laparoscopy combined with indocyanine green fluorescence imaging technique for hepatic cystic echinococcosis

**DOI:** 10.1186/s12893-020-00911-8

**Published:** 2020-10-22

**Authors:** Yu-Peng Li, Zhi-Gang Ma, Tuerhongjiang Tuxun, Zhi-De Li, Yuan Meng, Xiong Chen

**Affiliations:** 1 Department of Hepatobiliary Surgery, People’s Hospital of Xinjiang Uygur Autonomous Region, No. 91 Tianchi Road, Urumqi, 830000 Xinjiang Uygur China; 2 Liver Transplantation & Laparoscopic Surgery Department/Digestive & Vascular Surgery Center, The First Affiliated Hospital, Xinjiang Medical University, Urumqi, 830054 Xinjiang Uygur China

**Keywords:** Hepatic cystic echinococcosis, Laparoscopy, Pericystectomy, Indocyanine green (ICG), Fluorescence imaging, Boundary

## Abstract

**Background:**

With the mature application of laparoscopy in hepatobiliary surgery, laparoscopic treatment of hepatic cystic echinococcosis (CE) has made certain progress. But, due to the inherent limitations of laparoscopy and the growth characteristics of cystic echinococcosis, distinguishing the boundary between cystic lesion and normal hepatic parenchyma is pivotal importance for successful surgery. Indocyanine green (ICG) fluorescence imaging technology can view the boundary of lesion and normal tissue during the treatment of hepatic cystic echinococcosis. Applied laparoscopy combined with ICG fluorescence imaging technique for hepatic cystic echinococcosis may be an effective surgical strategy.

**Methods:**

The clinical data contained nine patients with hepatic cystic echinococcosis who underwent laparoscopic surgery with indocyanine green fluorescence imaging technique in authors’ institution from December 2018 to December 2019 were retrospectively analyzed. Indocyanine green was administered intravenously three days prior to surgery. The fluorescence acquisition system for real-time imaging was used during the surgery and the patients were followed up after surgery.

**Results:**

Of reported nine patients**,** six are male and the remaining three are female. The average age is (36.4 ± 7.6) years. For all subjects, surgical procedures were performed under laparoscopy with indocyanine green fluorescence system. This technique showed the clear boundary of the hepatic cyst with normal liver parenchyma. Total cystectomy in six patients, subtotal cystectomy in two patients and partial hepatectomy in one patient were performed respectively. The average operation time was 3.8 ± 0.9 h, blood loss 206.0 ± 120.7 ml. Neither blood transfusion nor post-operative complication was experienced. The average abdominal drainage time was 3.4 ± 0.9 days with hospital stay 5.7 ± 2.1 days. During the 6–12 months follow-up period, neither recurrence nor intraperitoneal implantation was found.

**Conclusions:**

Applied laparoscopy combined with ICG fluorescence imaging technique for hepatic cystic echinococcosis is safe and feasible. Enhanced boundary image can assist surgeons to complete radical resection and reduce complications.

## Background

Hepatic cystic echinococcosis is a zoonosis caused by the larval stage of *Echinococcus granulosus* and continues to be a public health threat in northwest China. Despite the sustained efforts to control and manage, the number of cystic echinococcosis patients are increasing [1]. After entering the alimentary tract, the parasite resides itself mainly in the liver. As the mostly targeted organ, hepatic cystic echinococcosis accounts for 75% of all human cases. The treatment approach is diversiform and stage dependent according to WHO-IWGE (World Health Organization-Informal Working Group on Echinococcosis) classification [2]. Radical resection is advocated whenever surgery is indicated. Up to now, pericystectomy and hepatectomy are the radical procedure with lower recurrence rate. However, these techniques require sophisticated hands and selective patients. Therefore, there are still space room for conservative surgery including subtotal cystectomy and endo-cystectomy [3]. With the introduction of laparoscopic technique, the safety, feasibility and efficacy of minimal invasive technique has been approved with acceptable mortality and morbidity.

The choice for laparoscopy is usually limited by the size, site, skills and intraoperative anatomy, especially the real boundary of lesion with the normal parenchyma [4]. Mostly, laparoscopic pericystectomy is converted to subtotal cystectomy or endo-cystectomy due to inaccurate identification of the boundary. Although, preoperative three-dimensional imaging would help surgeons to gain more detailed anatomical information, it would be more helpful if the boundary be identified intraoperatively. Previously, indocyanine green administration was reported to show the hepatocellular margins during laparoscopic resection [5]. However, no experience is reported the feasibility of indocyanine green administration in cystic echinococcosis patients undergoing laparoscopic resection. Herein, we report our single center experience laparoscopic resection of cystic echinococcosis with indocyanine green imaging and discuss its safety, feasibility as well as effectiveness along with surgical outcomes.

## Methods

### Clinical information

In current study, we have retrospectively analyzed the clinical data of nine patients who were diagnosed with hepatic cystic echinococcosis and received laparoscopic resection at Hepatobiliary Surgery Department of People's Hospital of Xinjiang Uygur Autonomous Region from December 2018 to December 2019. Routine cardio-pulmonary evaluation was carried out for all subjects. Serum analysis, abdominal ultrasound and computed tomography (CT) were also routinely carried out for the diagnosis and evaluation for lesion size, site and its correlation with major vessels. In patients with elevated serum bilirubin levels or bile duct dilation, MRI-cholangiography would be performed to determine whether there is biliary invasion or bile duct obstruction. The diagnosis for cystic echinococcosis was confirmed by postoperative pathology in all subjects. No patients were preoperatively administered with albendazole.

#### Configuration and injection of ICG

ICG (Brand name: Ruidu, SFDA approval number H20055881), specification 25 mg/piece, solvent is sterilized water for injection. Generally, it is injected intravenously in the periphery. Slowly and uniformly during the injection, closely observe the changes of the patient's heart rate, blood pressure, breathing, and oxygen saturation, and beware of allergic reactions. The injection timing is divided into preoperative injection and intraoperative injection.

Preoperative injection: 3 days before surgery, the dose is 0.5 mg/kg and the concentration is 5 mg/ml; Intraoperative injection: When the laparoscopic lens is inserted into the operation, the dose is 2.5 mg and the concentration is 2.5 mg/ml. When configured, the ICG powder is thoroughly mixed with the sterilized water for injection, and can be used for injection after it is completely dissolved. Note: The ICG solution should be freshly prepared. Do not use normal saline to dissolve the powder.

ICG fluorescence imaging: The fluorescence imaging system is an endoscopic fluorescence imaging system (PINPIONT), which consists of a video processor/light source (VPI), a camera, and a display. The camera irradiates the liver with excitation light at about 780 nm, and collects near-infrared light at about 840 nm. After processing by the host, the image is displayed.

#### Surgical methods

The patients were positioned in supine position and intubated, then 100 mg of hydrocortisone was administered intravenously to prevent possible allergic reactions. During the surgery, the central venous pressure was reduced to less than 5 cm of water column. The pneumoperitoneum was established and pressure maintained at 10–12 mmHg. The trocars were positioned corresponding to the size and site of the lesion. After thorough exploration, the small omental sac was dissected by using harmonic. The first hepatic portal was suspended ventricle drainage tube for hilar control whenever needed (Pringle method). Hepatic tissue was isolated from the gastrointestinal with hypertonic saline (100 g/kg) gauze. When the cystic wall is thin with high tension, the puncture device combined with echinococcosis rotary cutter was introduced decompress the cyst. Puncture site was carefully clipped or suture closed.

Pericystectomy is indicated for patients with single superficial lesion whose outer capsule wall thickness more than 3 mm and no major vascular involvement. Found the space between the outer capsule wall and liver tissue, hepatic parenchyma was dissected by using ultrasonic scalpel and CUSA under direct vision of PINPOINT system. Any vessels entering the lesion was clipped or sutured closed, and Endo-GIA was used whenever needed.

##### Subtotal cystectomy

Subtotal cystectomy was performed when the cyst was critically adjunct with major vasculatures. The space between cyst and hepatic parenchyma was identified following the PINPOINT system. The partial wall of the cyst was reserved aiming to avoid possible bleeding and / or leakage. Any larger biliary tracts or blood vessels entering the lesions was clipped whenever needed.

##### Partial hepatectomy

Due to the obvious fibrosis or atrophy of the liver tissue at the lesion site, it is located in the liver parenchyma, cause difficulties for hydatid cyst external cystectomy. Partial hepatectomy should be performed for cases involving invasion of liver important ducts. Use ultrasound knife combined with CUSA to cut off liver tissue, for larger bile ducts or blood vessels, titanium clips or bioclamps should be used for clamping, and Endo-GIA should be used for the second hepatic hilum.

##### ICG fluorescence imaging

The lesion boundary was clearly marked via PINPOINT system by using green fluorescence. The accurate site of the lesion and boundary with normal parenchyma was determined.

Routine abdominal drainage was practiced in all subjects and removed after surgery after the patients' liver function was normal, they were routinely given. Oral albendazole with dose of 20 mg/kg per day was administered for at least one month each course, for 6 consecutive courses of treatment with intervals of 7 to 10 days between each course of treatment.

#### Follow-up

Patients were followed up by telephone or outpatient after discharge. Ultrasound, routine blood test, liver function, medication status and any possible symptoms were carefully followed-up to June 30, 2020.

#### Statistics

IBM SPSS22.0 statistical software was used for data analysis. Quantitative data were expressed as mean ± standard deviation, qualitative data were used with rate description.

## Results

### Baseline information

**Fig. 1:**
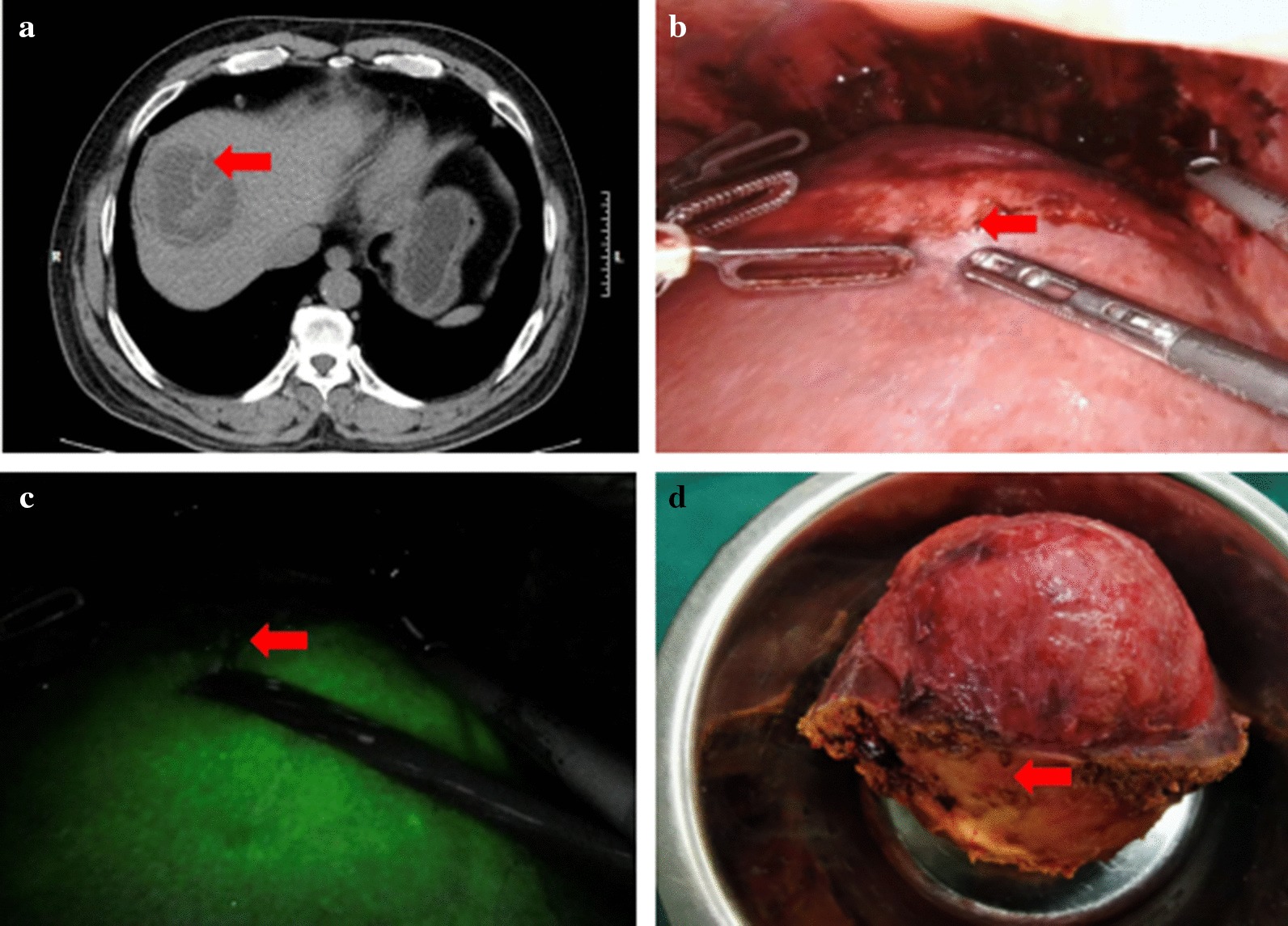
Computed tomography manifestation, intraoperative visual field and specimen of hepatic cystic hydatid. **a** Cystic echinococcosis in segment VIII(CE3a); **b** The arrow shows the border of hepatic cystic hydatid, the boundary is unclear to the naked eye; **c** The hepatic cystic echinococcosis lesion has no fluorescence display, a clear dividing line can be seen; **d** The surgical specimen of liver cystic hydatid focus, the arrow shows the external capsule of liver cystic hydatid focus

A total of 9 patients were included in this study, 6 males and 3 females. Five Uyghur, two Kazakh, one Han and one Tibet patients were enrolled current study. The average age of (36.4 ± 7.6) years. Five patients were overweight (BMI > 24), three patients were normal (18.5 ≤ BMI ≤ 24), one patient were malnutrition (BMI < 18.5). Serum albumin in 9 patients were normal (ALB ≥ 35 g/l). NRS2002 score showed that 1 patient had nutritional risk and 8 patients had no nutritional risk. For the patient at nutritional risk, oral nutrient solution was administered for 3–5 days preoperatively to improve nutritional status. All subjects were graded as Child–Pugh grade A and no patients have previous surgical history. All subjects have lived or ever travelled to affected area for cystic echinococcosis. The most frequent symptoms are intermittent upper abdominal pain in five patients, while, remaining four patients were identified during the routine physical examination. Of reported nine patients, five were positive and the rest four were negative. The preoperative abdominal ultrasonographical image and CT (Fig. 1a) indicated the diagnosis for cystic echinococcosis. The cysts were categorized according to the WHO/IWGE classification. There were 4 cases of multiple daughter cyst (CE2) and 5 cases of Ruptured cyst (CE3). The lesion is mainly located in the right lobe of the liver, with a maximum diameter of 9.4 ± 2.3 cm. Preoperative serum bilirubin levels were normal and no bile duct dilatation was observed on abdominal CT in 9 patients. So no MRI-cholangiography was done. The bile component can be seen in the cyst contents in some lesions, suggesting that it may occurs slightly bile leaks. (Table 1).

**Table 1:** General clinical information

Cases	Pathology (WHO-IWGE)	Lesion location	Maximum diameter (cm)	Preoperative infection/bile leakage	Operation method
1	CE2	S3	6	No	Pericystectomy
2	CE2	S5–6	9	No	Pericystectomy
3	CE2	S3–4	10	Bile leakage	Subtotal cystectomy
4	CE2	S7–8	10	Infection, bile leakage	Pericystectomy
5	CE3a	S5–8	8	Bile leakage	Pericystectomy
6	CE3a	S6–7	11	Bile leakage	Subtotal cystectomy
7	CE3b	S5–8	14	Infection, bile leakage	Right hemihepatectomy
8	CE3b	S5–6	10	Bile leakage	Pericystectomy
9	CE3a	S6	7	No	Pericystectomy

Two cases underwent subtotal cystectomy due to major vascular invasion in the liver. One case underwent right hemihepatectomy due to the first hepatic portal vascular invasion of the right liver

### Surgical parameters of patients

All patients successfully underwent laparoscopic surgery. Neither conversion to open laparotomy occurred nor allergic reaction caused by the overflow of hydatid cyst fluid during the operation was occurred; among them, 6 patients underwent laparoscopic pericystectomy, 2 patients underwent subtotal cystectomy and 1 patient underwent partial hepatectomy. The average operation time was 3.8 ± 0.9 h, t_average_ intraoperative bleeding was 206.0 ± 120.7 ml, and no blood transfusion occurred. Exploration of cutting edges resulted with small biliary leakage in four patients and laparoscopically sutured. No obvious bile leakage was observed, no T-tube was placed in the patients, and no complications such as bile leakage or bleeding occurred after surgery. The average value of serum ALT on the first day after surgery was 315.0 ± 89.5 (U/l) and the average value of serum AST was 283.3 ± 78.0 (U/l). The average time to return to normal was 4.5 ± 1.1 days. Six patients had hypoproteinemia after surgery (ALB < 35 g/l), and improved after strengthened oral nutrient solution. The patients carried drainage tube after operation for average 3.4 ± 0.9 days, and the average postoperative hospital stay was 5.7 ± 2.1 days. The patients were followed-up averagely for 9.7 ± 1.9 months. All of them completed the follow-up. None of the patients had recurrence of hepatic cystic echinococcosis or abdominal implantation.

### Results of fluorescence imaging of hepatic cystic echinococcosis

ICG was intravenously administered three days prior to surgery with no allergy. A weak circular fluorescent shadow was imaged in one CE3b patient, which was thought to as the patient’s lesion is huge and compress the surrounding bile ducts, which caused the obstruction of ICG excretion. In the remaining cases, their ICG metabolism was complete, and their normal liver and hydatid lesions showed no fluorescence.

**Fig. 2:**
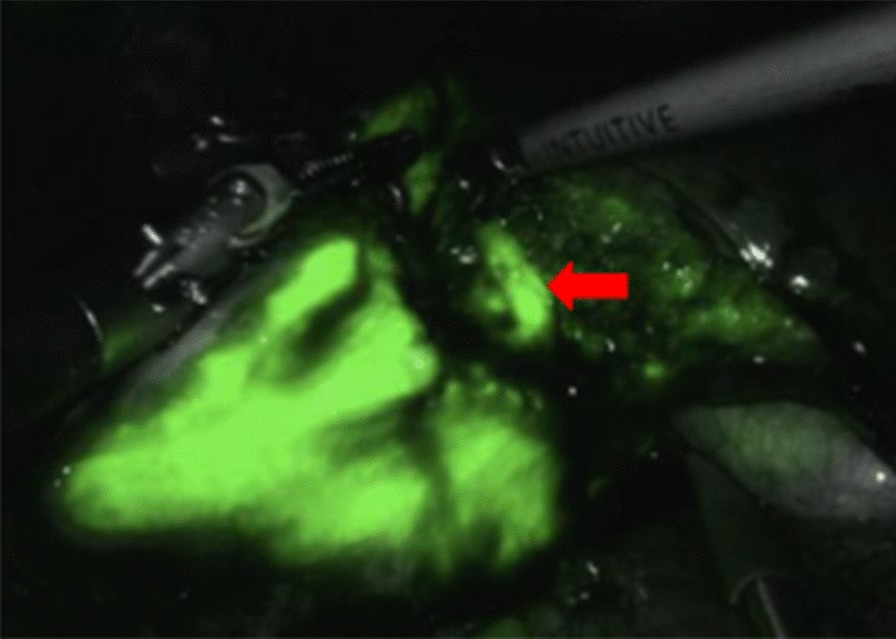
Surgical section under indocyanine green fluorescence imaging. The arrow shows normal liver tissue in section, no residual focus

All patients received intraperitoneal injection of ICG. From Fig. 1b, it can be seen that sometimes the boundary between cystic hydatid lesions and normal liver tissue is not very clear. From Fig. 1c, it can be seen that the hepatic cystic echinococcosis lesion has no fluorescence display, the image is black, and it will not change significantly with the extension of the ICG injection time. The surrounding liver tissue is green fluorescent. A clear dividing line can be seen between the two parts. If the lesion is located in the liver parenchyma, the fluorescence imaging cannot show the echinococcal lesion. After the liver parenchyma is disconnected, the light green fluorescence of the liver cut surface can be seen, and the liver hydatid lesion is still black. Observing the liver section and imaging after hepatic cystic echinococcosis lesion resection, no residual lesion was found (Fig. 2).

## Discussion

To the best of our knowledge, this is one of the few reports about laparoscopic management of hepatic cystic echinococcosis under the guidance of intraoperative ICG imaging. Our results showed that ICG imaging could help surgeon to find the space between the lesion and normal parenchymal lesion and facilitate surgical process.

With the mature application of laparoscopy in hepatobiliary surgery, laparoscopic treatment of hepatic cystic echinococcosis has made great progress and achieved good results [6]. The indications for laparoscopic surgery: (1) Hepatic cystic echinococcosis with single, multiple or collapsed inner cyst of hydatid cyst with mean diameter of > 5 cm (CE1–3). (2) The average diameter of hydatid cyst is ≤ 5 cm, but it is located in the first and second hepatic hilum of the liver, leading to serious complications, such as obstructive jaundice, portal hypertension, bougua syndrome, etc. (3) Hepatic cystic hydatid disease with serious complications (CE4, CE5). (4) Hepatic cystic hydatid with the average diameter < 5 cm, single cystic (CE1), multiple ascus (CE2) or collapsed inner cyst (CE3), could not adhere to drug therapy due to large adverse drug reactions, or the lesion continued to increase after more than half a year of drug therapy. The diameter of liver hydatid in the 9 patients in this study was greater than 5 cm, which was consistent with the surgical indications. In terms of preoperative evaluation, our experience with single medical centers is that ASA score are generally required I–III. It also requires that the patient's cardiopulmonary function be suitable for laparoscopic liver surgery. Since excessive obesity will affect the operating space of laparoscopic surgery, our center generally requires the BMI value below 29. Child–pugh grade of liver function is grade A or Grade B.

When endo-cystectomy is performed, the highest and weakest point of liver hydatid cyst wall is usually selected for puncture and suction decompression, as much as possible to prevent hydatid cyst fluid extravasation. The puncture device combined with echinococcosis rotary cutter is similar to Avtan’s device: Perforator Grinder-Aspirator Apparatus [7]. But the endo-cystectomy is related with higher incidence of residual cavity complications (8.28%–65.8%), recurrence rate (1.5%–36.6%), and reoperation rate (2.2%–3.2%) as well as biliary fistula (19.4%) compared to pericystectomy [8]. Endo-cystectomy is still one of the necessary methods of liver hydatid surgery in the following cases: (1) multiple surgeries, large lesions and narrow operating space. (2) The lesion adhered closely to the surrounding area and was difficult to be removed. (3) Single cystic hydatid with thin cyst wall and easy rupture, calcified cystic hydatid that is difficult to find in the gap, especially when hydatid cyst is close to the main blood vessels and bile ducts of hepatic hilum is difficult to separate.

As radical modality option, pericystectomy and hepatectomy hydatid significantly reduce the risk of postoperative complication [9]. Pericystectomy is indicated for patients with single superficial lesion whose outer capsule wall thickness more than 3 mm and no major vascular involvement. Between the outer cyst wall of a liver hydatid cyst and the liver parenchyma has a membrane-like fibrous structure called the “outer membrane”. The pathological results showed fibrosis of intrahepatic ducts around hepatic hydatid cysts, hyperplasia of surrounding fibrous tissues, and infiltration of lymphocytes. The immune reaction between liver hydatid lesions and the host is involved in the formation of the outer membrane, leading to the "disappearance" of liver cells around the hydatid and the formation of liver hydatid with biliary fistula, which is one of the main causes of death of liver hydatid. There is a potentially detachable gap between the hepatic hydatid cyst and the “outer membrane” along which the hepatic hydatid cyst can be completely removed. So, pericystectomy can reduce the recurrence of hepatic hydatid disease and complications of residual cavity, minimize the damage to liver parenchyma and reduce the risk of operation. The key to the success of the operation is to find the correct dissection space and correctly deal with the intrahepatic duct around hydatid. However, due to the inherent limitations of laparoscopy and the growth characteristics of cystic echinococcosis, distinguishing the boundary between cystic lesion and normal hepatic parenchyma is pivotal importance for successful surgery. Besides, failing to identify boundary may result in devastating rupture of the cyst and concurrent possible septic shock and implantation of the cystic lesion [10]. In recent years, with the application of ICG fluorescence imaging in laparoscopic surgery, laparoscopic liver tumor resection has realized visualization of tumor resection margins [11]. In current study, we have applied ICG fluorescence imaging technology to view the boundary of lesion and normal tissue during laparoscopic treatment of hepatic cystic echinococcosis. We observed that at the beginning of the surgery, after ICG was administered peripherally, a certain intensity of fluorescence could be observed in normal liver tissues after 3 to 5 min, but no fluorescence appeared in the cystic hydatid lesion tissue, and this part was shown as in black. With the extension of observation time, the fluorescence intensity of normal liver tissues of patients will become greener, but the lesion tissues have no obvious changes. Laparoscopic ICG fluorescence imaging can visualize the lesion boundary in real time, define the surgical margin, improve the rate of complete excision of the outer capsule, and reduce complications such as postoperative bile leakage and recurrence. When the surgeon dissects the liver along the vixulization ischemic line, even in the absence of clear markers in the liver parenchyma, the boundaries between the liver segments can be clearly shown inside the liver parenchyma, thereby reducing the need the stripping operation. Although ICG technology has many advantages, the use of laparoscopic ultrasound is still necessary in laparoscopic hepatic hydatid resection. Laparoscopic ultrasound can effectively judge the relationship between liver hydatid lesions and intrahepatic ducts, as well as the deformation of hepatic ducts, so as to reduce the incidence of important ducts damaged in the liver. For liver hydatid lesions with deep location, intraoperative ultrasound localization is required. Segmental liver staining with ICG technique is conducive to the implementation of anatomical partial liver resection, in which case intraoperative ultrasound is required to locate the target portal vein branches.

Partial hepatectomy also as the radical option for cystic echinococcosis. Some professors think that the total or partial pericystectomy should be considered the gold standard of treatment of hepatic cystic hydatid and the atypical hepatic resections are excessive for a benign pathology, except in cases in which the cyst completely replaces an entire hepatic lobe [12]. In our report, one patient who’s liver hydatid lesions occupied 4 segments of the right liver performed right hemihepatectomy. From our single-center experience, partial hepatectomy was preferred when hepatic hydatid cyst was confined to a liver segment or liver lobe, or when hepatic hydatid cyst had severe peripheral hepatobiliary invasion and was likely to have a large long-term biliary leakage in the surgical stump. Liver transplantation is an important method for the treatment of advanced hepatic echinococcosis [13]. Hepatic cystic hydatid lesions compress the hepatic hilum, resulting in serious complications, which cannot be effectively treated by traditional treatment methods, autologous liver transplantation should be performed. Allogeneic liver transplantation is performed when there is a risk of liver failure after autologous transplantation.

Intraoperative hemorrhage is a common cause of conversion from laparoscopic hepatectomy to laparotomy, especially hepatic venous hemorrhage which is difficult to control. When the liver was congested or the liver section bleeding, the fluorescence intensity would be weak and would not serve as a good guide. Low central venous pressure can effectively reduce bleeding from intraoperative hepatic venous lacerations, and we usually maintain a central venous pressure of 0–5 cm of water column during the surgery. When this method is performed, no obvious blood will emerge from the ethmoidal orifices of the hepatic veins, and hemostasis can be stopped by compression with a hemostatic sponge. Undetectable blood vessels closely attached to liver hydatid lesions usually performed subtotal cystectomy which retain part of the liver hydatid outer capsule and can avoid haemorrhage. Small traffic vessels can be closed directly with energy instruments, while larger vessels need to be closed with biofilms or ligated with wires. For the branches of hepatic veins, it is necessary to fully dissect and dissociate, realize full dimensional naked, and confirm the diameter, course, and geometric relationship with the main hepatic vein and hepatic hydatid lesions. Then, at the site of implementation, the vessel clamp is used for exact clipping and disconnection, or it is directly disconnected with the linear cutting ligator. Avoid cutting blood vessels without adequate exposure with an ultrasound knife or cutting them in half with a blood vessel clamp. Besides, intermittent use the Pringle method to block the first hepatic portal can effectively control intraoperative bleeding. Some researchers think that Pringle method should be avoided. Only the hepatic pedicle branch of the resected liver should be blocked, so as to reduce the ischemia reperfusion injury of the reserved liver tissue. But it is not easy to implement, and there is still a risk of bleeding when dissecting the area of hepatic pedicle. Prophylactic administration of dexamethasone static injection at the beginning of the operation and strict control of the blocking time can reduce hepatic ischemia–reperfusion injury.

Bile leakage is one of the common complications after surgery and severely affects the postoperative recovery of patients, and even requires indwelling bile duct T-tube drainage during operation, sometime, causing the patients need long-term T-ub [14]. MRI-cholangiography can be used to evaluate the biliary tract defect or obstruction before surgery, but cannot find microbial leakage and cannot be dynamically indicated during operation. Since ICG is excreted from the body through bile metabolism, that can help determine bile leakage during surgery. At the same time, the ICG fluorescence fusion image can show the biliary tract movement and the existence of mutations, which has a certain effect on reducing biliary tract injury during the operation. In addition, the application of ICG fluorescence imaging can find other imaging techniques or lesions that cannot be found by inspection and palpation, which can effectively reduce the postoperative recurrence of the lesions [15]. In laparoscopic metastatic liver cancer resection, ICG fluorescence imaging combined with intraoperative ultrasound detection is significantly better than preoperative CT or intraoperative ultrasound in detecting lesions smaller than 3 mm [16]. In this group of cases, we performed intraoperative ICG fluorescence examination of the liver section without residual lesions. In the imaging review performed after operation, and no lesion remained. This method achieves the goal of radical resection, avoids residual lesions, and benefits for the patients.

However, we also recognize that ICG fluorescence development also has certain limitations: (1) The penetration depth of the fluorescent tissue emitted by ICG is only 5–10 mm, so for deeper hydatid lesions, intraoperative ultrasound should be used in combination. (2) The uptake and distribution of ICG in the liver are affected by many factors. The diffusion imaging of the fluorescence boundary is common, and the recirculation can also reduce the fluorescence difference in the liver area. (3) The imaging effect of ICG is affected by many factors such as liver function status, so, it is difficult to choose the precise time and dose of administration according to the specific condition of the patient.

This study retrospectively analyzed the data of 9 patients with hepatic cystic echinococcosis who underwent laparoscopic combined ICG. This method may improve the opportunity of radical resection of the disease and reduce the incidence of common intraoperative and postoperative complications.

## Conclusions

This study illustrates the surgical technique and short and long-term outcomes of indocyanine green fluorescence imaging during laparoscopic management of hepatic cystic echinococcosis patients. Also this study was limited by the number of cases, we could found that this surgical technique assisted surgeons to complete resection of hepatic hydatid, especially the pericystectomy of hepatic hydatid by enhanced boundary image. Because of the magnification of the laparoscope and the good development of the biliary tract by fluorescence imaging, it was beneficial to reduce the complication of biliary leakage and improved the prognosis of patients.

## Supplementary information

**Additional file 1.** Additional clinical information.

## Data Availability

All data generated or analysed during this study are included in this published article. Additional clinical information are given in Additional file 1.

## Abbreviations

ICG: Indocyanine green
CE: Cystic echinococcosis
NRS 2002: European Nutrition Risk Screening 2002
WHO-IWGE: World Health Organization-Informal Working Group on Echinococcosis
CT: Computed tomography
S: Liver segmentation, according to Couinaud
ALT: Alanine aminotransferase
AST: Aspartate aminotransferase
