# Supplementary material for: The application of laparoscopy combined with indocyanine green fluorescence imaging technique for hepatic cystic echinococcosis
Source: BMC Surg. 2020 Oct 22;20:249. doi: 10.1186/s12893-020-00911-8 (PMC7579955; doi:10.1186/s12893-020-00911-8)
Supplement: Supplementary file 1 — Additional file 1. Additional clinical information. [file 12893_2020_911_MOESM1_ESM.docx]

**Additional clinical information**

**Table S1 Preoperative clinical data**

| Cases | Gender | Age | BMI | ALB | Child-Pugh | Pathology | Maximum diameter (cm) | Lesion location |
| --- | --- | --- | --- | --- | --- | --- | --- | --- |
| 1 | 1 | 2 | 26.1 | 42 | A | CE2 | 6 | S3 |
| 2 | 1 | 3 | 23.5 | 42 | A | CE2 | 9 | S5-6 |
| 3 | 1 | 3 | 25.3 | 39 | A | CE2 | 10 | S3-4 |
| 4 | 1 | 4 | 27.7 | 38 | A | CE2 | 10 | S7-8 |
| 5 | 1 | 3 | 26.6 | 42 | A | CE3a | 8 | S5-8 |
| 6 | 1 | 1 | 22.5 | 40 | A | CE3a | 11 | S6-7 |
| 7 | 2 | 4 | 25.7 | 39 | A | CE3b | 14 | S5-6-7-8 |
| 8 | 2 | 4 | 23.5 | 38 | A | CE3b | 10 | S5-6 |
| 9 | 2 | 1 | 18.0 | 36 | A | CE3a | 7 | S6 |

**(In the age category, the number 1 represents the age range from 21 to30, 2: the age range from 31to 40, 3: the age range from 41to 50)**

**Table S2 Intraoperative and postoperative clinical data**

| Cases | Operation method | Operation time(hours) | Surgical bleeding(ml) | ALT | AST | Drainage tube days | Postoperative days |
| --- | --- | --- | --- | --- | --- | --- | --- |
| 1 | Pericystectomy | 3 | 50 | 280 | 250 | 2 | 3 |
| 2 | Pericystectomy | 4 | 255 | 325 | 285 | 3 | 4 |
| 3 | Subtotal cystectomy | 3 | 120 | 190 | 175 | 3 | 4 |
| 4 | Pericystectomy | 5 | 380 | 370 | 330 | 4 | 9 |
| 5 | Pericystectomy | 4 | 200 | 320 | 280 | 4 | 7 |
| 6 | Subtotal cystectomy | 3 | 150 | 240 | 220 | 5 | 7 |
| 7 | Right hemihepatectomy | 5 | 400 | 500 | 450 | 4 | 8 |
| 8 | Pericystectomy | 4.5 | 200 | 350 | 310 | 3 | 5 |
| 9 | Pericystectomy | 3 | 100 | 260 | 250 | 3 | 4 |
